# Two-Dimensional GeC/MXY (M = Zr, Hf; X, Y = S, Se) Heterojunctions Used as Highly Efficient Overall Water-Splitting Photocatalysts

**DOI:** 10.3390/molecules29122793

**Published:** 2024-06-12

**Authors:** Guangzhao Wang, Wenjie Xie, Sandong Guo, Junli Chang, Ying Chen, Xiaojiang Long, Liujiang Zhou, Yee Sin Ang, Hongkuan Yuan

**Affiliations:** 1 School of Electronic Information Engineering, Key Laboratory of Extraordinary Bond Engineering and Advanced Materials Technology of Chongqing, Yangtze Normal University, Chongqing 408100, China; xwj52739820@163.com (W.X.); longxiaojiang@yznu.edu.cn (X.L.); 2 School of Electronic Engineering, Xi’an University of Posts and Telecommunications, Xi’an 710121, China; sandongyuwang@163.com; 3 School of Physical Science and Technology, Southwest University, Chongqing 400715, China; jlchang66@126.com; 4 School of Electronic and Information Engineering, Anshun University, Anshun 561000, China; ychenjz@163.com; 5 School of Physics, University of Electronic Science and Technology of China, Chengdu 610054, China; ljzhou86@uestc.edu.cn; 6 Science, Mathematics and Technology, Singapore University of Technology and Design, Singapore 487372, Singapore

**Keywords:** hybrid density functional, GeC/MXY heterojunctions, direct Z-scheme, photocatalysis, water-splitting

## Abstract

Hydrogen generation by photocatalytic water-splitting holds great promise for addressing the serious global energy and environmental crises, and has recently received significant attention from researchers. In this work, a method of assembling GeC/MXY (M = Zr, Hf; X, Y = S, Se) heterojunctions (HJs) by combining GeC and MXY monolayers (MLs) to construct direct Z-scheme photocatalytic systems is proposed. Based on first-principles calculations, we found that all the GeC/MXY HJs are stable van der Waals (vdW) HJs with indirect bandgaps. These HJs possess small bandgaps and exhibit strong light-absorption ability across a wide range. Furthermore, the built-in electric field (BIEF) around the heterointerface can accelerate photoinduced carrier separation. More interestingly, the suitable band edges of GeC/MXY HJs ensure sufficient kinetic potential to spontaneously accomplish water redox reactions under light irradiation. Overall, the strong light-harvesting ability, wide light-absorption range, small bandgaps, large heterointerfacial BIEFs, suitable band alignments, and carrier migration paths render GeC/MXY HJs highly efficient photocatalysts for overall water decomposition.

## 1. Introduction

In recent years, environmental pollution and shortages of non-renewable energy have become increasingly severe. Photocatalytic water decomposition (PCWD) for hydrogen generation is considered as an effective approach to alleviating the energy crisis and environmental pollution [1,2,3,4,5,6,7,8,9,10]. In 1972, TiO${\;\;}_{2}$ was first used as a photocatalyst (PC) to decompose water into hydrogen and oxygen [11]. The PCWD process typically comprises three steps: light capture, photoinduced carrier separation/transfer, and water redox reactions occurring on the surfaces of the catalysts [12,13,14]. The required water-splitting PC (WSPC) must have a band edge exceeding the water redox level; specifically, the H^+^/H_2_ and H_2_O/O_2_ levels must fall between the valence band maximum (VBM) and conduction band minimum (CBM) [15,16,17]. Consequently, the bandgap of a WSPC for PCWD should be greater than 1.23 eV. Furthermore, considering the energy loss during the process of photoinduced carriers transferring to the catalyst’s surfaces and the kinetic potentials required to drive the water redox reactions, the bandgap of the WSPC is typically required to be greater than 1.8 eV [12,18]. In addition to the bandgap requirement, the activity of WSPCs strongly depends on other factors such as photostability, light capture ability, trapping of and photoinduced carrier recombination, and the catalyst’s surface reactivities towards the hydrogen/oxygen evolution reaction (HER/OER) [19]. Although researchers have developed a series of PCs; a few single WSPCs simultaneously possess the advantages of wide light response extent, good carrier mobility, high photoexcited carrier separation, spatially separated reaction sites, strong redox capacity, and lower overpotentials for the HER and OER processes. Thus, it is still urgent to explore new photocatalytic mechanisms and develop highly efficient WSPCs.

Inspired by photosynthesis in green plants, a direct Z-scheme mechanism was constructed to overcome the shortcomings of single WSPCs [20,21,22]. A typical direct Z-scheme WSPC is usually composed of two parts: the hydrogen production PC (HPPC) and oxygen production PC (OPPC) [23,24]. The photoinduced electrons and holes recombine at the interface between the HPPC and OPPC, resulting in remnant electrons at the HPPC and excess holes at the OPPC. This process leads to the efficient spatial separation of photogenerated carriers, thus obtaining strong redox capacity to drive water-splitting. Up to now, the direct Z-scheme mechanism has been experimentally realized in a series of composites, including TiO${\;\;}_{2}$/ZnIn_2_S_4_ [25], aza-CMP/C_2_N, [26], Cd_0.5_Zn_0.5_/BiVO${\;\;}_{4}$ [27], α-Fe_2_O_3_/g-C_3_N_4_ [28], black P/BiVO${\;\;}_{4}$ [29], CdS/MoS${\;\;}_{2}$ [30], CdS/Co_1−*x*_S [31], and TiO${\;\;}_{2}$/CuO${\;\;}_{2}$ [32]. In particular, direct Z-scheme two-dimensional (2D) van der Waals (vdW) heterojunction (HJ) PCs (HJPCs) exhibit excellent photocatalytic performance due to their highly specific surface area, abundant active sites, good carrier mobility, and tunable interfaces [26,33,34]. In addition, strong electron–hole coupling and charge transfer around such heterointerfaces have been experimentally observed [35,36,37] and theoretically proposed [38,39,40,41,42]. This facilitates interlayer carrier recombination and helps to achieve the Z-scheme photocatalytic mechanism.

The graphene-like hexagonal GeC monolayer (ML) receives considerable attention due to its excellent electronic, mechanical, magnetic, and optical properties [43,44,45,46]. Especially, it possesses lower stiffness and a bigger Poisson’s ratio compared to graphene [47]. Therefore, the excellent characteristics of the GeC ML promote it to achieve device applications in the fields of electronics, optoelectronics, and photovoltaic [48]. More excitingly, GeC thin films have been experimentally synthesized by the chemical vapor deposition (CVD) [49] and laser ablation [50] methods. Since it is known that a large variety of 2D layers can be fabricated using the mechanical exfoliation and CVD [51,52,53] methods, we can speculate that the GeC ML may also be synthesized using similar preparation methods. The GeC ML not only has a stable plane structure, but also shares a similar honeycomb structure and lattice constants with many other 2D materials; thus, some GeC-based HJs have been designed and studied [54,55,56,57,58,59,60,61,62,63]. Although many literature works have explored the photocatalytic performance of the GeC ML and GeC-based type-II HJPCs, there are still relatively few reports on the direct Z-scheme mechanism of GeC-based HJs, which remains an open question thus far. It is interesting and meaningful to find suitable 2D materials to construct direct Z-scheme HJPCs with GeC MLs. Recently, MXY (M = Zr, Hf; X, Y = S, Se) MLs with a stable 1T phase have been demonstrated to exhibit excellent mechanical, thermal, thermoelectric, piezoelectricity, optical, and catalytic properties [64,65,66,67,68,69,70]. In particular, HfS${\;\;}_{2}$, HfSe${\;\;}_{2}$, ZrS${\;\;}_{2}$, and ZrSe${\;\;}_{2}$ MLs have been experimentally verified [71,72,73,74,75], as well as Janus MoSSe and WSSe, which have been experimentally synthesized [76,77,78]. We can speculate that the Janus HfSSe and ZrSSe could be potentially fabricated by selenizing HfS${\;\;}_{2}$ (or HfSe${\;\;}_{2}$) and ZrS${\;\;}_{2}$ (or ZrSe${\;\;}_{2}$) MLs, respectively, using the CVD method, which is similar to the method used for synthesizing MoSSe and WSSe MLs. Moreover, the photocatalytic properties of MXY-based HJs have also been explored [79,80,81,82,83,84,85,86]. However, the photocatalytic properties of HJs composed of GeC and MXY MLs have not been reported yet. Therefore, we expect to combine GeC MLs and MXY MLs to construct highly efficient direct Z-scheme HJPCs.

Theoretical calculation is a simple and effective way to screen and design potential direct Z-scheme WSPCs [87,88,89,90,91]. From density functional theory (DFT) calculations, one can determine whether a type-II HJ exhibits a direct Z-scheme or type-II photocatalytic mechanisms based on the carrier migration path, judged according to the built-in electric field (BIEF) direction [28,92]. If the BIEF promotes interlayer carrier recombination, the carrier transfer belongs to a Z-scheme mechanism. Otherwise, the type-II mechanism dominates. Herein, first-principles calculations are performed to explore the possibility of constructing GeC/MXY HJs using GeC and MXY MLs as direct Z-scheme systems. Work functions (Φ) and charge density differences (CDDs) indicate that the BIEFs promote all eight GeC/MXY HJs to form the Z-scheme photocatalytic mechanism for overall water-splitting. Furthermore, all these HJs possess strong visible light-absorption capacity and substantial near-infrared light-absorption capacity. Moreover, these HJs can provide sufficient driving forces to overcome the HER and OER overpotentials to perform overall water redox reactions. These results are expected to guide experiment progress in exploring 2D direct Z-scheme WSPCs.

## 2. Results and Discussion

Before constructing GeC/MXY HJs, the geometric and electronic properties of GeC and MXY MLs are first investigated. The corresponding structural models for GeC, ZrS${\;\;}_{2}$, ZrSe${\;\;}_{2}$, ZrSSe, HfS${\;\;}_{2}$, HfSe${\;\;}_{2}$, and HfSSe MLs are plotted in Figure S1. The obtained $E_{g}$ values for GeC, ZrS${\;\;}_{2}$, ZrSe${\;\;}_{2}$, ZrSSe, HfS${\;\;}_{2}$, HfSe${\;\;}_{2}$, and HfSSe MLs are, respectively, 2.87, 2.02, 1.19, 1.46, 2.13, 1.33, and 1.56 eV, and the corresponding lattice parameters are, respectively, 3.235, 3.685, 3.800, 3.743, 3.645, 3.768, and 3.705 Å (see Table 1). Furthermore, the bond lengths of Ge–C in GeC, Zr–S in ZrS${\;\;}_{2}$, Zr–Se in ZrSe${\;\;}_{2}$, Zr–S (or Zr–Se) in ZrSSe, Hf–S in HfS${\;\;}_{2}$, Hf–Se in HfSe${\;\;}_{2}$, and Hf–S (or Hf–Se) in HfSSe are 1.868, 2.574, 2.706, 2.568 (or 2.713), 2.552, 2.685, and 2.550 (or 2.687) Å, respectively (see Table 1). GeC possesses a direct bandgap with both the VBM and CBM located at the *K* point, while all the MXY MLs are indirect bandgap semiconductors with the VBM and CBM, respectively, located at the Γ and *M* points (see Figure S2). All these results agree well with previous reports [54,55,56,57,58,64,65,66,67,93], as displayed in Table 1, indicating that our calculations are reliable.

**Figure 1:**
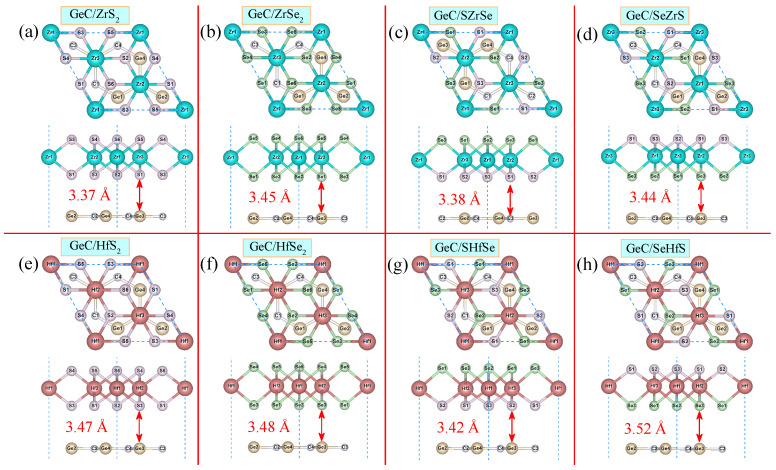
Top and side views for various GeC/MXY HJs.

Although the lattice constants of GeC and MXY are obviously different, the 2 × 2 GeC supercell could match well with the $\sqrt{3}$ × $\sqrt{3}$ MXY supercell. Herein, we define the lattice mismatch as [2 × $|L_{\mathrm{sGeC}}-L_{\mathrm{sMXY}}|$/$\left(L_{\mathrm{sGeC}}+L_{\mathrm{sMXY}}\right)$] × 100%, where $L_{\mathrm{sGeC}}$ and $L_{\mathrm{sMXY}}$ are the lattice constants for the GeC and MXY supercells, respectively. The calculated lattice mismatches between GeC and MXY to construct various GeC/MXY HJs are 1.38%, 1.71%, 0.20%, 0.20%, 2.47%, 0.87%, 0.82%, and 0.82%, respectively. These small lattice mismatches are favorable for the direct growth of GeC/MXY HJs by CVD or physical epitaxy [94]. Considering that the ZrSSe (or HfSSe) ML possesses two different surfaces, we loaded $\sqrt{3}$ × $\sqrt{3}$ ZrS${\;\;}_{2}$, ZrSe${\;\;}_{2}$, ZrSSe, HfS${\;\;}_{2}$, HfSe${\;\;}_{2}$, and HfSSe MLs onto a 2 × 2 GeC ML to construct eight different GeC/MXY HJs, i.e., GeC/ZrS${\;\;}_{2}$, GeC/ZrSe${\;\;}_{2}$, GeC/SZrSe, GeC/SeZrS, GeC/HfS${\;\;}_{2}$, GeC/HfSe${\;\;}_{2}$, GeC/SHfSe, and GeC/SeHfS. The corresponding models of GeC/MXY HJs are shown in Figure 1. Note that, here, the average of the lattice parameters for GeC and MXY is used to build GeC/MXY HJs, and the lattice parameters for GeC/MXY HJs are illustrated in Table 2.

The thermodynamic stability of GeC/MXY HJs is assessed by calculating the interface formation energies ($E_{f}$) as follows:

(1) $$E_{f}=\left(E_{\mathrm{GeC}/\mathrm{MXY}}^{T}-E_{\mathrm{GeC}}^{T}-E_{\mathrm{MXY}}^{T}\right)/S,$$

where $E_{\mathrm{GeC}/\mathrm{MXY}}^{T}$, $E_{\mathrm{GeC}}^{T}$, and $E_{\mathrm{MXY}}^{T}$, respectively, denote the total energies of GeC/MXY HJs, GeC ML, and MXY ML. The calculated $E_{f}$ values for all the considered HJs are negative, which means that the construction of all these GeC/MXY HJs release heat and tend to be thermodynamically stable. The $E_{f}$ values in Table 2 range from −29.4 to −18.5 meV/Å${\;\;}^{2}$, suggesting that these HJs are formed via the interaction between vdW and the MLs [95]. Moreover, the interlayer distances of GeC/MXY HJs vary from 3.367 to 3.519 Å (see Table 2), aligning with the results of some other typical vdW structures [96,97,98,99,100]. Consequently, all the examined GeC/MXY structures are classified as vdW HJs. The interfacial formation energy can be directly defined as: $E_{f}$ = $E_{\mathrm{GeC}/\mathrm{MXY}}^{T}-E_{\mathrm{GeC}}^{T}-E_{\mathrm{MXY}}^{T}$. In this case, the $E_{f}$ values for GeC/ZrS${\;\;}_{2}$, GeC/ZrSe${\;\;}_{2}$, GeC/SZrSe, GeC/SeZrS, GeC/HfS${\;\;}_{2}$, GeC/HfSe${\;\;}_{2}$, GeC/SHfSe, and GeC/SeHfS HJs are −0.66, −1.04, −1.05, −1.07, −0.89, −1.04, −1.00, and −1.03 eV, respectively. The $E_{f}$ value of the GeC/SZrSe (or GeC/SHfSe) HJ is sightly more negative than that of the GeC/SeZrS (or GeC/SeHfS) HJ, indicating that the formation of the GeC/SZrSe (or GeC/SHfSe) HJ is energetically slightly more favorable. During experimental preparation, both GeC/SZrSe (or GeC/SHfSe) and GeC/SeZrS (or GeC/SeHfS) HJs are likely to be prepared. The difference in their preparation lies in the fact that the Janus ZrSSe (or HfSSe) ML contacts the GeC ML with different surfaces.

**Figure 2:**
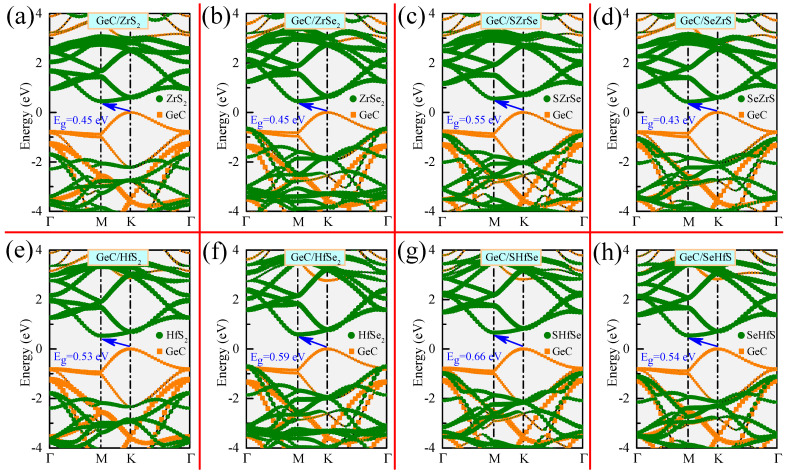
Band structures for various GeC/MXY HJs. The orange (or green) dots denote the contribution from the GeC (or MXY) layer.

The band structures for various GeC/MXY HJs, computed using the HSE06 hybrid functional, are illustrated in Figure 2. The orange color denotes the contribution from the GeC layer, while the green color represents the contribution from the MXY layers. All the GeC/MXY HJs are indirect bandgap semiconductors, as their VBMs are located at the *K* point, while the CBMs are positioned at the *M* point. The corresponding bandgaps for GeC/MXY HJs are 0.45 (0.446), 0.45 (0.453), 0.55, 0.43, 0.53, 0.59, 0.66, and 0.54 eV, respectively (see Table 3), which are significantly lower than those of the corresponding MLs. Consequently, these HJs are expected to achieve high solar energy utilization. The CBMs originate from the MXY layer, whereas the VBMs come from the GeC layer, confirming the staggered type-II nature of all the examined GeC/MXY HJs. These facilitate the spatial separation of the photoinduced carriers. Furthermore, the band alignments of the GeC and MXY layers in the HJs retain the primary characteristic of their isolated MLs, suggesting that the vdW interaction at the heterointerface does not significantly influence the electronic properties of the layers.

**Figure 3:**
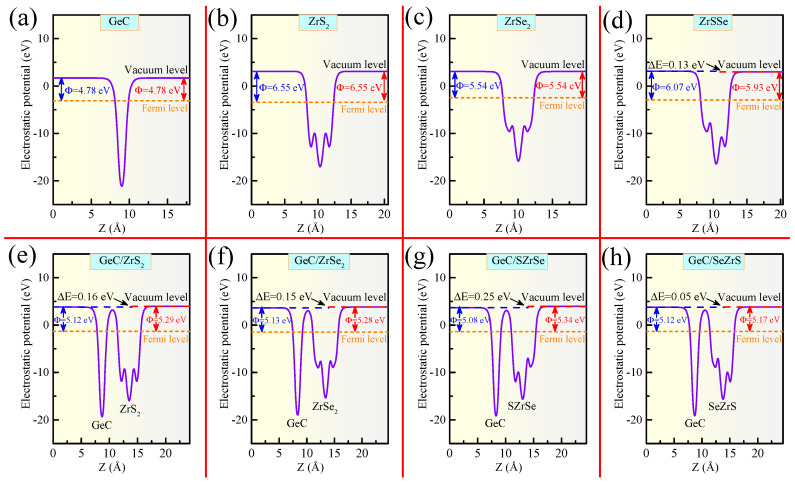
Electrostatic potential diagrams of (**a**) GeC, (**b**) ZrS${\;\;}_{2}$, (**c**) ZrSe${\;\;}_{2}$, (**d**) ZrSSe, (**e**) GeC/ZrS${\;\;}_{2}$, (**f**) GeC/ZrSe${\;\;}_{2}$, (**g**) GeC/SZrSe, and (**h**) GeC/SeZrS, respectively.

The work function (Φ) and CDD are crucial in determining the BIEF direction at the heterointerface, a factor that holds a decisive role in the design of Z-scheme PCs [101]. The Φ values can be obtained as follows:

(2) $$\Phi =E_{\mathrm{vac}}-E_{F},$$

where $E_{\mathrm{vac}}$ and $E_{F}$ refer to the vacuum and Fermi energy levels, respectively. The electrostatic potentials (EPs) of the relative MLs and HJs are depicted in Figure 3 and Figure S3. The vacuum levels of the two surfaces in GeC, ZrS${\;\;}_{2}$, ZrSe${\;\;}_{2}$, HfS${\;\;}_{2}$, and HfSe${\;\;}_{2}$ are identical, meaning that the electrostatic potential differences (EPDs) (ΔE) between the two sides are all zero. The corresponding Φ values for these MLs are 4.80, 6.55, 5.54, 6.46, and 5.68 eV, respectively. The difference in the electronegativity between the S and Se atoms at the two opposing sides of the Janus ZrSSe and HfSSe results in inherent BIEFs perpendicular to the plane, causing the vacuum levels on both surfaces to differ. Consequently, the work functions are naturally distinct on the surfaces of both ZrSSe and HfSSe. The corresponding ΔE values are 0.13 and 0.11 eV for ZrSSe and HfSSe, respectively. The Φ values for the S-side (Se-side) for ZrSSe and HfSSe are 6.07 (5.93) and 5.95 (5.84) eV, respectively. Evidently, GeC exhibits a lower Φ value compared to the MXY MLs. Once GeC and MXY come into contact to form a GeC/MXY HJ, electrons will transfer from the material with a lower work function to the one with a higher work function until dynamic equilibrium is achieved. Consequently, a BIEF is established across the GeC/MXY heterointerface, pointing from GeC towards MXY. Additionally, the vacuum levels on both sides of the GeC/MXY HJs also differ. The calculated values ΔE for the various GeC/MXY HJs are 0.16, 0.15, 0.25, 0.05, 0.09, 0.10, 0.20, and 0.02 eV, respectively. Furthermore, the work functions for the respective GeC/MXY HJs are 5.12 (5.29), 5.13 (5.28), 5.08 (5.34), 5.12 (5.17), 5.06 (5.14), 5.05 (5.15), 5.00 (5.20), and 5.04 (5.06) eV. This indicates that, when GeC and MXY contact to form a GeC/MXY HJ, electrons migrate from GeC to MXY to reach the same Fermi level.

**Figure 4:**
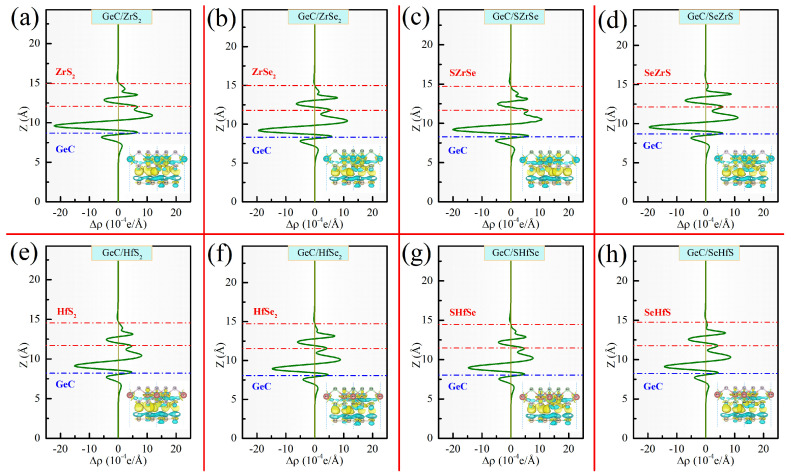
VCDDs and PACDDs for various GeC/MXY HJs.

Moreover, we analyzed the charge transfer at the heterointerface region in GeC/MXY HJs by calculating the visual charge density difference (VCDD) based on the following relationship [102,103]:

(3) $$\Delta \rho =\rho_{\mathrm{GeC}/\mathrm{MXY}}-\rho_{\mathrm{GeC}}-\rho_{\mathrm{MXY}},$$

where $\rho_{\mathrm{GeC}/\mathrm{MXY}}$, $\rho_{\mathrm{GeC}}$, and $\rho_{\mathrm{MXY}}$ represent the charge densities for the GeC/MXY HJ, GeC ML, and MXY ML, respectively. The yellow (or cyan) region denotes charge accumulation (or consumption). Additionally, the planar-averaged CDD (PACDD) along the *z*-direction is obtained by the following equation [103,104]:

(4) $$\Delta \rho \left(z\right)=\int \rho_{\mathrm{GeC}/\mathrm{MXY}}\mathrm{dxdy}-\int \rho_{\mathrm{GeC}}\mathrm{dxdy}-\int \rho_{\mathrm{MXY}}\mathrm{dxdy},$$

where $\int \rho_{\mathrm{GeC}/\mathrm{MXY}}\mathrm{dxdy}$, $\int \rho_{\mathrm{GeC}}\mathrm{dxdy}$, and $\int \rho_{\mathrm{MXY}}\mathrm{dxdy}$ represent the planar-averaged charge densities of the GeC/MXY HJ, GeC ML, and MXY ML, respectively. The positive (or negative) value indicates the charge accumulation (or consumption). It can be clearly seen from Figure 4 that the charge around the heterointerfaces of all the GeC/MXY HJs is redistributed. Charge accumulation primarily occurs at the heterointerface region near MXY, while charge consumption mainly takes place at the heterointerface region near GeC. This further confirms that electrons migrate from GeC to MXY in all the GeC/MXY HJs. The Bader charge analysis also suggests that 0.11, 0.09, 0.11, 0.09, 0.08. 0.07, 0.08, and 0.07 *e*, respectively, migrate from GeC to MXY in the various GeC/MXY HJs. The charge transfer at the heterointerfaces of GeC/MXY HJs could cause the BIEF to point away from GeC toward MXY, which commonly promotes the spatial separation of carriers, thus extending the lifetime of photoexcited carriers and enhancing the photocatalytic activity.

**Figure 5:**
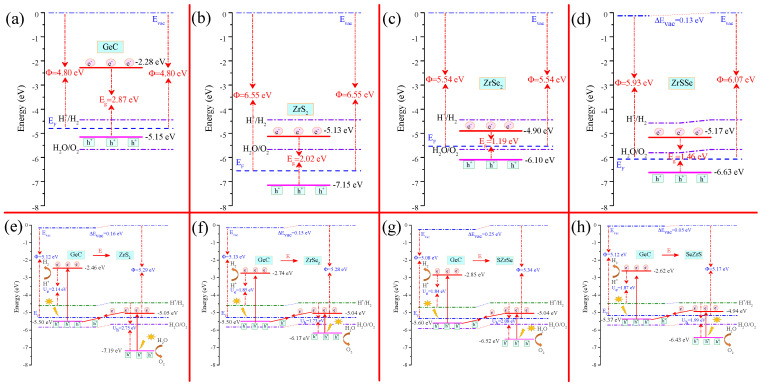
Band alignments for (**a**) GeC, (**b**) ZrS${\;\;}_{2}$, (**c**) ZrSe${\;\;}_{2}$, (**d**) ZrSSe, (**e**) GeC/ZrS${\;\;}_{2}$, (**f**) GeC/ZrSe${\;\;}_{2}$, (**g**) GeC/SZrSe, and (**h**) GeC/SeZrS versus vacuum level.

As is well known, the type-II band alignment corresponds to both the type-II and direct Z-scheme photocatalytic mechanisms based on different charge transfer pathways. For a type-II HJPC, the band edges of its two components must simultaneously straddle the water redox potentials. Thus, a type-II HJPC usually does not provide sufficient driving force for water redox processes. For a direct Z-scheme HJPC, the VBM of one component should be lower than the water oxidation potential (WOP), while the CBM of the other component should be higher than the water reduction potential (WRP) [105,106]. Thus, a direct Z-scheme HJPC is usually capable of proving sufficient driving force for redox reactions. Next, we arrange the band edges of the GeC ML, MXY MLs, and GeC/MXY HJs in contrast to the water redox levels in Figure 5 and Figure S4, in order to further determine the photocatalytic mechanisms of the considered GeC/MXY HJs. It is known that the water redox levels are determined by the electrochemical potentials relative to the vacuum level, so the difference in the vacuum energy levels on the two surfaces of PCs causes the movement of H${\;\;}^{+}$/H${\;\;}_{2}$ and H_2_O/O_2_ levels between the two surfaces. The band edges of GeC only span the WOP, indicating that GeC is only suitable for the HER. Conversely, the band edges of MXY MLs solely cross the WRP, meaning that MXY MLs only serve for the OER. Thus, neither GeC nor MXY alone could achieve overall PCWD.

Since the photocatalytic mechanisms for all GeC/MXY HJs are similar, we will use GeC/ZrS_2_ as an illustrative example for a detailed discussion. As the GeC ML and ZrS_2_ ML approach each other to form the GeC/ZrS_2_ HJ, electrons migrate from GeC to ZrS_2_ due to the smaller work function of GeC compared to ZrS_2_. Consequently, the GeC and ZrS_2_ layers become positively and negatively charged, respectively. This results in a BIEF that is directed away from GeC toward ZrS_2_ across the GeC/ZrS_2_ heterointerface. Electrons in GeC are repelled by the negatively charged ZrS_2_, causing GeC’s bands to bend upward. Similarly, ZrS_2_’s bands will bend downward near the heterointerface due to the same mechanism [107,108]. To simplify the discussion, we have omitted the band bending in the band-alignment diagram of GeC/MXY HJs. When exposed to sunlight, both GeC and ZrS_2_ can absorb photons with greater energy than their respective bandgaps. This stimulates electrons to transition from the valence bands (VBs) to the conduction bands (CBs), leaving holes in the VBs. However, GeC is unsuitable for the OER due to its higher VBM than the WOP, while ZrS_2_ is unsuitable for the HER owing to its lower CBM than the WRP. This implies that the photoinduced holes in the VBs of GeC (or the photoexcited holes in the CBs of ZrS_2_) cannot directly participate in the OER (or HER) process. The calculated conduction band offset (CBO) and valence band offset (VBO) are 2.59 and 1.70 eV, respectively (see Table 3). Due to the BIEF directed from GeC to ZrS_2_, the migration of photoinduced electrons from the CBs of GeC to the CBs of ZrS_2_ and the migration of photoinduced holes from the VBs of ZrS${\;\;}_{2}$ to the VBs of GeC are hindered. Conversely, the photoexcited electrons are encouraged to migrate from the CBs of ZrS${\;\;}_{2}$ to the VBs of GeC, where they recombine with the photoexcited holes. Furthermore, the interlayer bandgap of 0.45 eV is significantly smaller than both the CBO and the VBO, favoring the interlayer electron–hole recombination. Consequently, GeC (or ZrS${\;\;}_{2}$) accumulates more photoinduced electrons (or holes). Naturally, the superfluous electrons on the CBs for GeC can achieve the HER, while the excess holes on the VBs of ZrS${\;\;}_{2}$ can realize the OER. Evidently, the migration path of photoexcited carriers is like a “Z”. Thus, the GeC/ZrS${\;\;}_{2}$ HJ constitutes a direct Z-scheme system. The spatial separation of photoexcited electrons and holes contributes to enhancing photocatalytic efficiency. Additionally, schematic diagrams illustrating the photocatalytic mechanisms of all considered MLs and HJs versus the normal hydrogen electrode (NHE) are presented in Figures S5 and S6.

Furthermore, the sufficient kinetic potentials ($U_{e}$ and $U_{h}$) provided by photoexcited electrons and holes are crucial for driving the OERs and HERs. The $U_{e}$ and $U_{h}$ values affect the number of active electrons and holes participating in water redox reactions, thereby influencing the photocatalytic activity. Here, $U_{e}$ (or $U_{h}$) is defined as the potential difference between the CBM and the H${\;\;}^{+}$/H${\;\;}_{2}$ level (or between the H${\;\;}^{+}$/H${\;\;}_{2}$ level and the VBM). Given that the water redox levels depend on the pH values, $U_{e}$ and $U_{h}$ can be expressed as follows [109]:

(5) $$\begin{array}{c} U_{e}=U_{e}\left(pH=0\right)-pH\times 0.059\;V,\\ U_{h}=U_{h}\left(pH=0\right)+pH\times 0.059\;V. \end{array}$$

**Figure 6:**
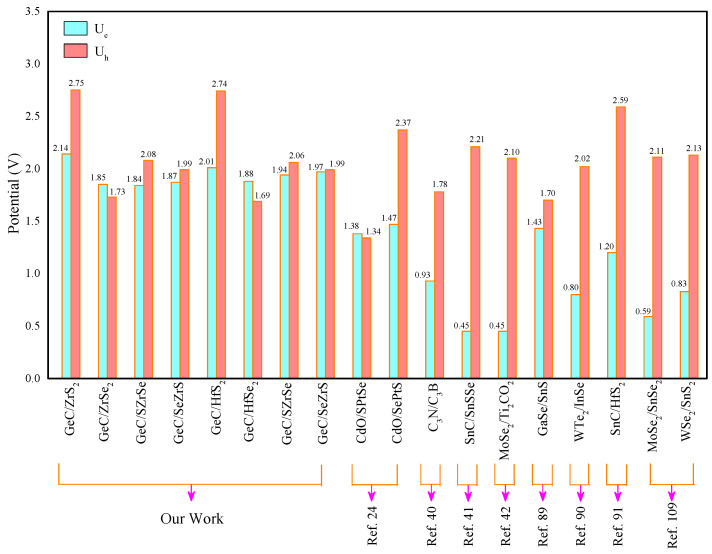
Comparison of $U_{e}$ and $U_{h}$ values of the proposed GeC/MXY HJs with some other reported HJs [24,40,41,42,89,90,91,109].

For the sake of simplicity, we will only discuss the $U_{e}$ (or $U_{h}$) value at pH = 0. The calculated $U_{e}$ and $U_{h}$ values are 2.14 and 2.75 V, respectively, which are comparable to some previously studied Z-scheme PCs (see Figure 6) [24,40,41,42,89,90,91,109]. Consequently, GeC/ZrS${\;\;}_{2}$ HJ emerges as a highly efficient Z-scheme WSPC.

The CBO (VBO) values for GeC/ZrSe${\;\;}_{2}$, GeC/SZrSe, GeC/SeZrS, GeC/HfS${\;\;}_{2}$, GeC/HfSe${\;\;}_{2}$, GeC/SHfSe, and GeC/SeHfS HJs are 2.30 (0.68), 2.19 (0.92), 2.32 (1.07), 2.34 (1.79), 2.18 (0.71), 2.15 (0.99), and 2.28 (1.12) eV, respectively (see Table 3). Obviously, the bandgaps of these HJs are smaller than their CBOs and VBOs, which is conducive to interlayer electron–hole recombination. Additionally, the BIEF direction is pointing from GeC to MXY. Similarly, the GeC/ZrS${\;\;}_{2}$, GeC/ZrSe${\;\;}_{2}$, GeC/SZrSe, GeC/SeZrS, GeC/HfS${\;\;}_{2}$, GeC/HfSe${\;\;}_{2}$, GeC/SHfSe, and GeC/SeHfS HJs are all Z-scheme WSPCs. Moreover, the obtained $U_{e}$ ($U_{h}$) values are 1.85 (1.73), 1.84 (2.08), 1.87 (1.99), 2.01 (2.74), 1.88 (1.69), 1.94 (2.06), and 1.97 (1.99) V, respectively (see Table 3). These values are also close to those reported for Z-scheme WSPCs (see Figure 6) [24,40,41,42,89,90,91,109]. This indicates that the GeC/ZrSe${\;\;}_{2}$, GeC/SZrSe, GeC/SeZrS, GeC/HfS${\;\;}_{2}$, GeC/HfSe${\;\;}_{2}$, GeC/SHfSe, and GeC/SeHfS HJs could supply sufficient dynamic potentials to drive HERs and OERs under light irradiation.

**Figure 7:**
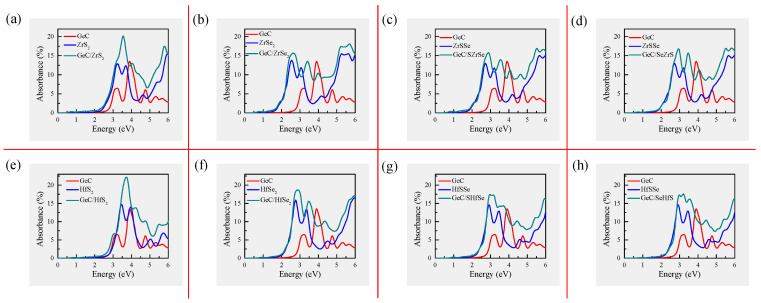
(**a**–**h**) Optical absorption curves of various GeC/MXY HJs compared with those of GeC and MXY MLs.

At the initial stage of photocatalytic water-splitting, the light absorption capacity serves as another crucial factor. For highly efficient solar utilization, a wide and intense light absorption spectrum is typically required. Therefore, we investigated the optical absorption curves of GeC, MXY, and GeC/MXY HJs using the HSE06 method. The optical absorption coefficient can be calculated using the following formula [110]:

(6) $$\alpha \left(\omega \right)=\frac{\sqrt{2}\omega }{c}{\left[\sqrt{\epsilon_{1}^{2}\left(\omega \right)+\epsilon_{2}^{2}\left(\omega \right)}-\epsilon_{1}\left(\omega \right)\right]}^{1/2},$$

where $\epsilon_{1}$ (or $\epsilon_{2}$) represents the real (or imaginary) part of the dielectric function and ω is the frequency of light. As shown in Figure 7, GeC/MXY HJs possess strong visible light absorption ability and non-negligible near-infrared light absorption ability. In addition, GeC/MXY HJs exhibit higher absorption coefficients in the visible and near-infrared light regions, along with a redshift of the absorption spectra, compared to the corresponding MLs. Herein, the GeC/MXY HJs demonstrate excellent light absorption capacity. Furthermore, the proper band alignments and suitable directions of the heterointerface BIEF enable these GeC/MXY HJs to form a Z-scheme photocatalytic mechanism. This facilitates the HERs and OERs to occur in different sublayers and provides sufficient driving force to spontaneously achieve water redox reactions under illumination. Generally speaking, GeC/XYs HJs are promising candidates for direct Z-scheme WSPCs.

## 3. Computational Details

In this work, the GeC/MXY (M = Zr, Hf; X, Y = S, Se) HJs are constructed by stacking the $\sqrt{3}$ × $\sqrt{3}$ MXY supercell onto a 2 × 2 GeC supercell with an 18 Å vacuum layer to eliminate the image interaction between adjacent layers. Additionally, dipole correction is introduced along the z-direction [111]. All DFT calculations were carried out using *VASP5.4* [112,113], and the electron–ion interactions were described using the projector-enhanced wave (PAW) method [114]. The generalized gradient approximation (GGA) [115] of Perdew–Burke–Ernzerhof (PBE) [116] was employed for the exchange correlation functional. Furthermore, Grimme’s DFT-D3 [117,118] method was employed to account for weak vdW interactions. The Monkhorst–Pack k-point grid for the first Brillouin zone was set to 13 × 13 × 1 (or 7 × 7 × 1) for MLs (or HJs). The energy cutoff was set to 500 eV, and all structures were sufficiently optimized with an energy (or force) tolerance of 10${\;\;}^{-5}$ eV (10${\;\;}^{-2}$ eV/Å). Given that GGA-PBE tends to underestimate the bandgaps [119], the Heyd–Scuseria–Ernzerhof functional (HSE06) was applied to accurately compute the electronic and optical properties [120]. The optical absorption spectra were computed based on the imaginary part of the dielectric functional, following the Kramers–Kronig dispersion relationship [110], and the band alignments of MLs and HJs were referenced to a common vacuum level.

## 4. Conclusions

In summary, the potential applications of GeC/MXY (M = Zr, Hf; X, Y = S, Se) HJs have been investigated through the calculation of their geometric, electronic, optical properties, band arrangement, and interface binding energies. Based on first-principles calculations, we analyzed their photocatalytic mechanism. All the considered GeC/MXY HJs, namely GeC/ZrS${\;\;}_{2}$, GeC/ZrSe${\;\;}_{2}$, GeC/SZrSe, GeC/SeZrS, GeC/HfS${\;\;}_{2}$, GeC/HfSe${\;\;}_{2}$, GeC/SHfSe, and GeC/SeHfS, were found to be direct Z-scheme photocatalytic systems with band edges spanning the water redox potentials. Charge redistribution at the heterointerface results in the formation of a BIEF pointing from GeC to MXY, enhancing the separation of the photoinduced carriers. Excitingly, the GeC/MXY HJs exhibit strong redox capacity for photocatalytic water decomposition, ensuring that the HER and OER processes occur spontaneously under light irradiation. Furthermore, the GeC/MXY HJs demonstrated strong visible light absorption and some near-infrared light absorption, guaranteeing efficient utilization of solar energy. These theoretical findings indicate that these GeC/MXY HJs are all promising WSPCs.

## Figures and Tables

**Table 1:** Lattice constants (*a*), bond lengths ($L_{B}$), bandgaps ($E_{g}$), dipole moments (μ), and EPDs (ΔE) between two sides for GeC and MXY MLs.

Systems	*a* (Å)	*a* (Å) (Refs.)	$L_{B}$ (Å)	$L_{B}$ (Å) (Refs.)	$E_{g}$ (eV)	$E_{g}$ (eV) (Refs.)	μ (D)	ΔE (eV)
GeC	3.235	3.26 [54,55], 3.233 [56]	1.868	1.882 [54], 1.887 [56]	2.87	3.01 [54], 2.90 [55]	0	0
		3.263 [57]				2.88 [56], 2.782 [57]		
						2.85 [58]		
ZrS${\;\;}_{2}$	3.685	3.70 [64,65], 3.69 [67]	2.574	2.58 [67], 2.570 [93]	2.02	1.99 [67], 1.96 [93]	0	0
		3.669 [93]						
ZrSe${\;\;}_{2}$	3.800	3.82 [64,65], 3.75 [67]	2.706	2.69 [67], 2.702 [93]	1.19	1.07 [67], 1.14 [93]	0	0
		3.786 [93]						
ZrSSe	3.743	3.73 [67]	2.568 (2.713)	2.55 (2.72) [67]	1.46	1.37 [67]	0.043	0.135
HfS${\;\;}_{2}$	3.645	3.66 [64,65], 3.65 [66]	2.552	2.55 [66], 2.56 [67]	2.13	2.03 [67], 2.07 [93]	0	0
		3.65 [67], 3.628 [93]		2.548 [93]				
HfSe${\;\;}_{2}$	3.768	3.82 [64], 3.78 [65,66]	2.685	2.69 [66], 2.68 [67]	1.33	1.16 [67], 1.26 [93]	0	0
		3.72 [67], 3.751 [93]		2.681 [93]				
HfSSe	3.705	3.71 [66], 3.68 [67]	2.550 (2.687)	2.55 (2.69) [66], 2.54 (2.69) [67]	1.56	1.45 [67]	0.035	0.110

**Table 2:** Lattice parameters (*a*), interlayer distances ($d_{i}$), interface formation energies ($E_{f}$), dipole moments (μ), EPDs (ΔE) between two surfaces, and charge transferred from GeC layer (ΔQ) in various GeC/MXY HJs.

Systems	*a* (Å)	$d_{i}$ (Å)	$E_{f}$ (meV/Å${\;\;}^{2}$)	μ (D)	ΔE (eV)	ΔQ (*e*)
GeC/ZrS${\;\;}_{2}$	6.426	3.367	−18.5	0.16	0.16	0.11
GeC/ZrSe${\;\;}_{2}$	6.527	3.446	−28.1	0.15	0.15	0.09
GeC/SZrSe	6.477	3.376	−28.8	0.24	0.25	0.11
GeC/SeZrS	6.477	3.436	−29.4	0.04	0.05	0.09
GeC/HfS${\;\;}_{2}$	6.392	3.468	−25.2	0.08	0.09	0.08
GeC/HfSe${\;\;}_{2}$	6.499	3.484	−28.5	0.10	0.10	0.07
GeC/SZrSe	6.444	3.421	−27.8	0.19	0.20	0.08
GeC/SeZrS	6.444	3.519	−28.5	0.01	0.02	0.07

**Table 3:** The bandgap ($E_{g}$), CBO, VBO, $U_{e}$, and $U_{h}$ values for GeC/MXY HJs.

Systems	$E_{g}$ (eV)	CBO (eV)	VBO (eV)	$U_{e}$ (V)	$U_{h}$ (V)
GeC/ZrS${\;\;}_{2}$	0.45	2.59	1.70	2.14	2.75
GeC/ZrSe${\;\;}_{2}$	0.45	2.30	0.68	1.85	1.73
GeC/SZrSe	0.55	2.19	0.92	1.84	2.08
GeC/SeZrS	0.43	2.32	1.07	1.87	1.99
GeC/HfS${\;\;}_{2}$	0.53	2.34	1.79	2.01	2.74
GeC/HfSe${\;\;}_{2}$	0.59	2.18	0.71	1.88	1.69
GeC/SHfSe	0.66	2.15	0.99	1.94	2.06
GeC/SeHfS	0.54	2.28	1.12	1.97	1.99

## Data Availability

The original contributions presented in the study are included in the article/Supplementary Material, further inquiries can be directed to the corresponding authors.

## Supplementary Materials

The following supporting information can be downloaded at: https://www.mdpi.com/article/10.3390/molecules29122793/s1, Figure S1: Top and side view of optimized geometries of GeC, ZrS${\;\;}_{2}$, ZrSe${\;\;}_{2}$, ZrSSe, HfS${\;\;}_{2}$, HfSe${\;\;}_{2}$ and HfSSe, respectively; Figure S2: Band structures of GeC, ZrS${\;\;}_{2}$, ZrSe${\;\;}_{2}$, ZrSSe, HfS${\;\;}_{2}$, HfSe${\;\;}_{2}$ and HfSSe, respectively; Figure S3: Electrostatic potential diagrams of GeC, HfS${\;\;}_{2}$, HfSe${\;\;}_{2}$, HfSSe, GeC/HfS${\;\;}_{2}$, GeC/HfSe${\;\;}_{2}$, GeC/SHfSe and GeC/SeHfS, respectively; Figure S4: Schematic diagrams of the photocatalytic mechanisms for GeC, HfS${\;\;}_{2}$, HfSe${\;\;}_{2}$, HfSSe, GeC/HfS${\;\;}_{2}$, GeC/HfSe${\;\;}_{2}$, GeC/SHfSe and GeC/SeHfS versus vacuum level; Figure S5: Schematic diagrams of the photocatalytic mechanisms for GeC, ZrS${\;\;}_{2}$, ZrSe${\;\;}_{2}$, ZrSSe, GeC/ZrS${\;\;}_{2}$, GeC/ZrSe${\;\;}_{2}$, GeC/SZrSe and GeC/SeZrS versus NHE; Figure S6: Schematic diagrams of the photocatalytic mechanisms for GeC, HfS${\;\;}_{2}$, HfSe${\;\;}_{2}$, HfSSe, GeC/HfS${\;\;}_{2}$, GeC/HfSe${\;\;}_{2}$, GeC/SHfSe and GeC/SeHfS versus NHE; Table S1: POSCAR file for the optimized GeC/ZrS${\;\;}_{2}$; Table S2: POSCAR file for the optimized GeC/ZrSe${\;\;}_{2}$; Table S3: POSCAR file for the optimized GeC/SZrSe; Table S4: POSCAR file for the optimized GeC/SeZrS; Table S5: POSCAR file for the optimized GeC/HfS${\;\;}_{2}$; Table S6: POSCAR file for the optimized GeC/HfSe${\;\;}_{2}$; Table S7: POSCAR file for the optimized GeC/SHfSe; Table S8: POSCAR file for the optimized GeC/SeHfS.
