# Supplementary material for: Two-Dimensional GeC/MXY (M = Zr, Hf; X, Y = S, Se) Heterojunctions Used as Highly Efficient Overall Water-Splitting Photocatalysts
Source: Molecules. 2024 Jun 12;29(12):2793. doi: 10.3390/molecules29122793 (PMC11206627; doi:10.3390/molecules29122793)
Supplement: Supplementary file 1 [file molecules-29-02793-s001.zip › molecules-2959122-supplementary.pdf]

Supporting Information

Two-Dimensional GeC/MXY (M = Zr, Hf; X, Y = S, Se) Heterojunctions Used as Highly Efficient Overall Water-Splitting Photocatalysts

Guangzhao Wang[†1,*], Wenjie Xie[†1], Sandong Guo[2], Junli Chang[3], Ying Chen[4], Xiaojiang Long[1], Liujiang Zhou[5], Yee Sin Ang[6,*], Hongkuan Yuan[3,*]

[1]School of Electronic Information Engineering, Key Laboratory of Extraordinary Bond Engineering and Advanced Materials Technology of Chongqing, Yangtze Normal University, Chongqing 408100, China.

[2]School of Electronic Engineering, Xi'an University of Posts and Telecommunications, Xi'an 710121, China.

[3]School of Physical Science and Technology, Southwest University, Chongqing 400715, China.

[4]School of Electronic and Information Engineering, Anshun University, Anshun 561000, China.

[5]School of Physics, University of Electronic Science and Technology of China, Chengdu 610054, China.

[6]Science, Mathematics and Technology, Singapore University of Technology and Design, Singapore 487372, Singapore.

† These authors contributed equally to this work

[*] Corresponding author. E-mail address: wangyan6930@yznu.edu.cn/wangyan6930@126.com (Guangzhao Wang), yeesin_ang@sutd.edu.sg (Yee Sin Ang), yhk10@swu.edu.cn (Hongkuan Yuan)

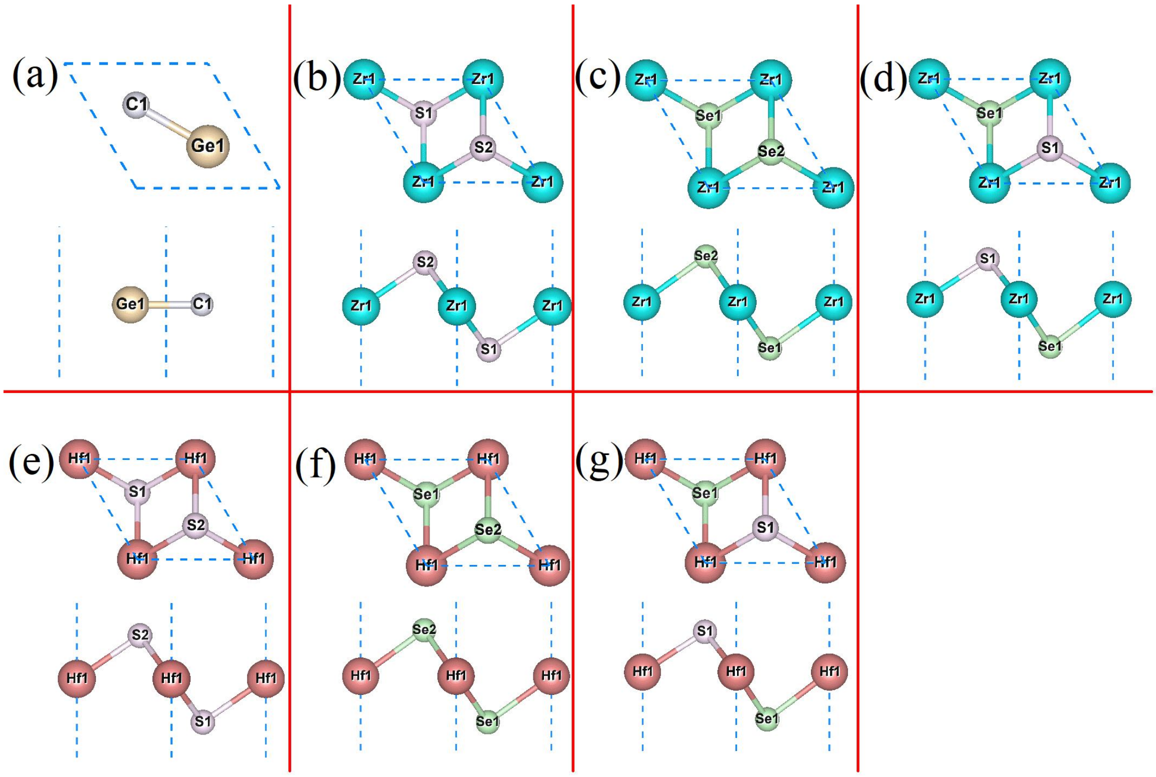

Fig. S1 Top and side view of optimized geometries of (a) GeC, (b) ZrS_2, (c) $ZrSe_2$, (d) ZrSSe, (e) HfS_2, (f) $HfSe_2$ and (g) HfSSe, respectively.

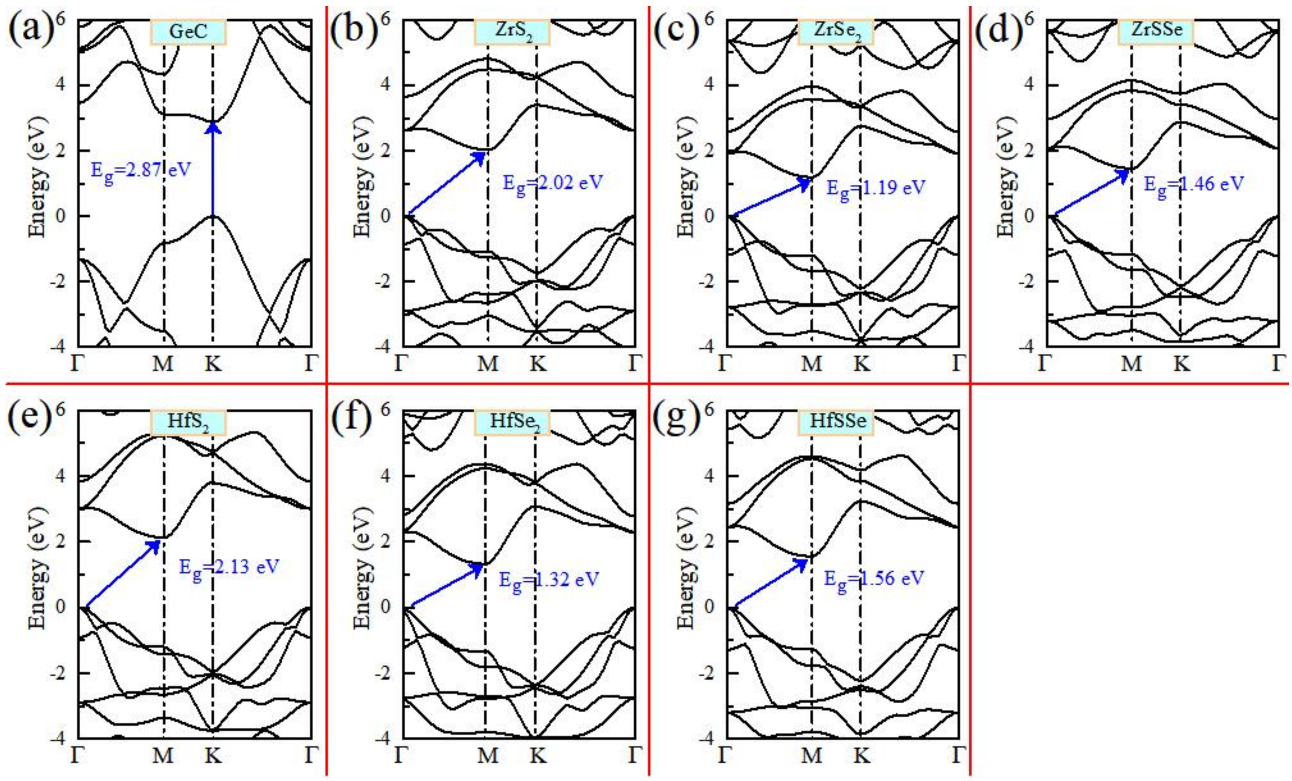

Fig. S2 Band structures of (a) GeC, (b) ZrS_2, (c) $ZrSe_2$, (d) ZrSSe, (e) HfS_2, (f) $HfSe_2$ and (g) HfSSe, respectively.

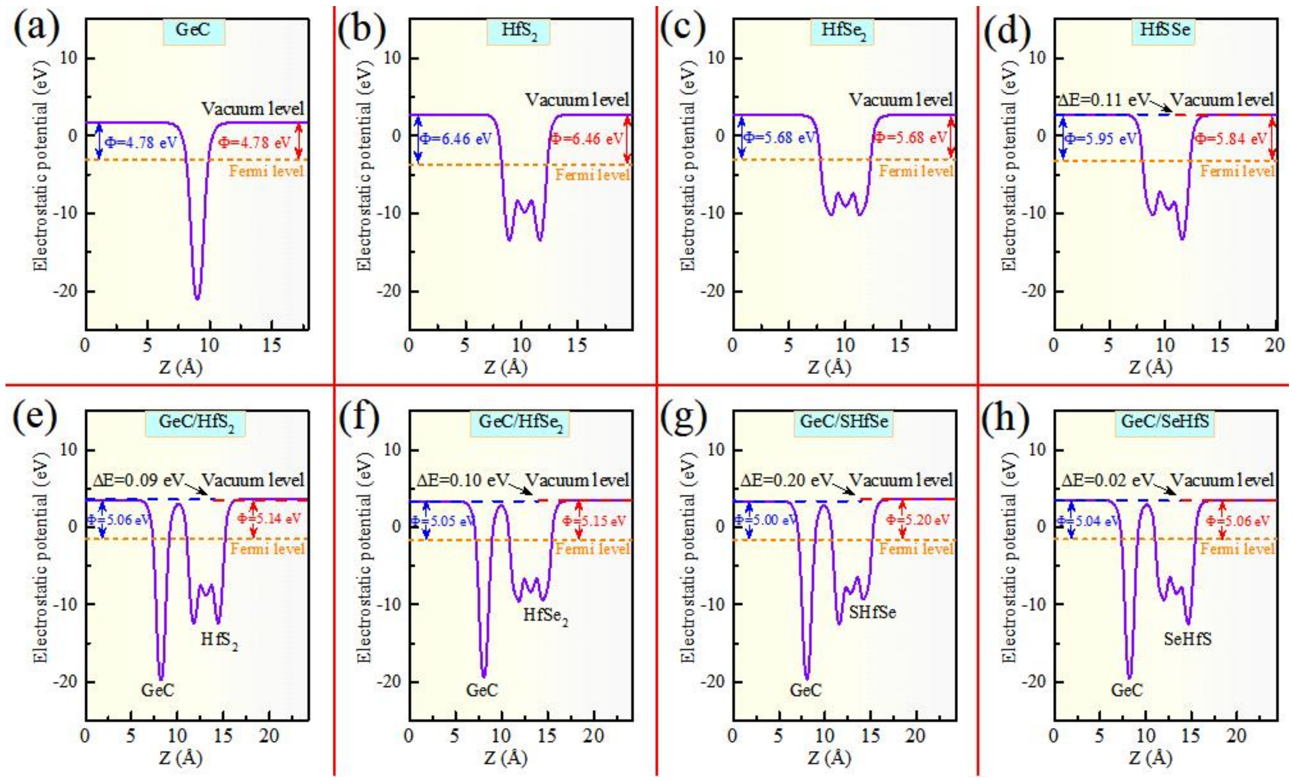

Fig. S3 Electrostatic potential diagrams of (a) GeC, (b) HfS_2, (c) $HfSe_2$, (d) HfSSe, (e) GeC/HfS_2, (f) GeC/$HfSe_2$, (g) GeC/SHfSe and (h) GeC/SeHfS, respectively.

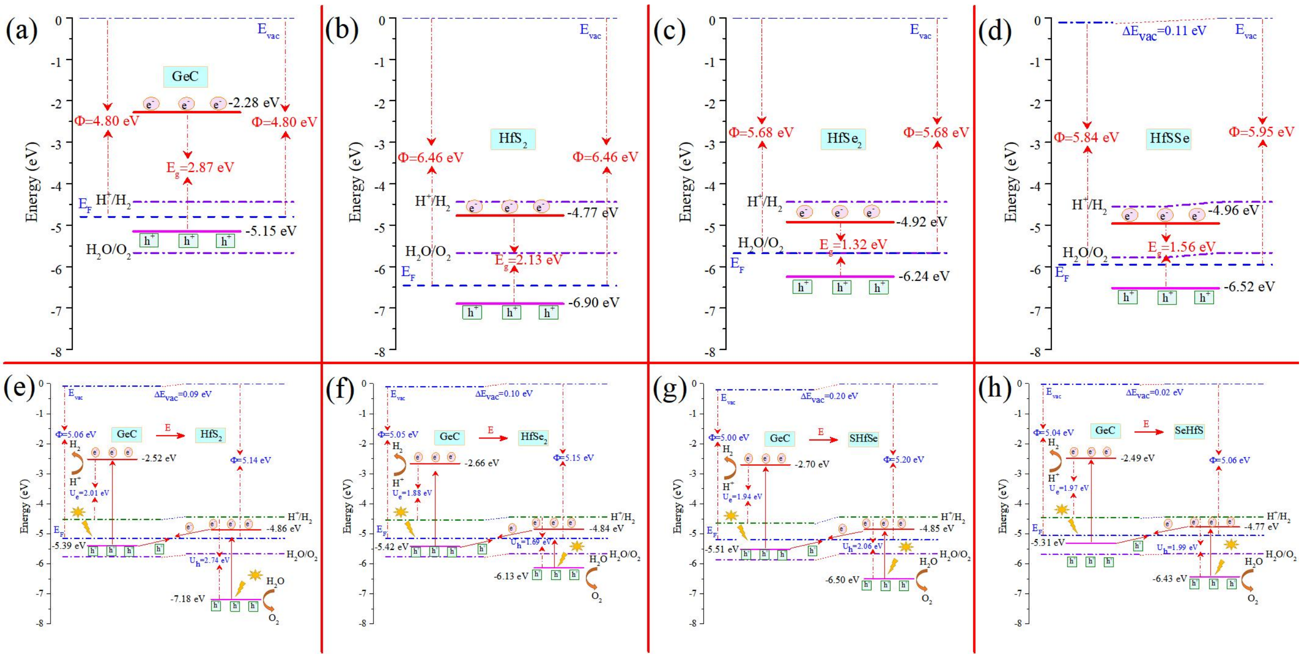

Fig. S4 Schematic diagrams of the photocatalytic mechanisms for (a) GeC, (b) HfS_2, (c) $HfSe_2$, (d) HfSSe, (e) GeC/HfS_2, (f) GeC/$HfSe_2$, (g) GeC/SHfSe and (h) GeC/SeHfS versus vacuum level.

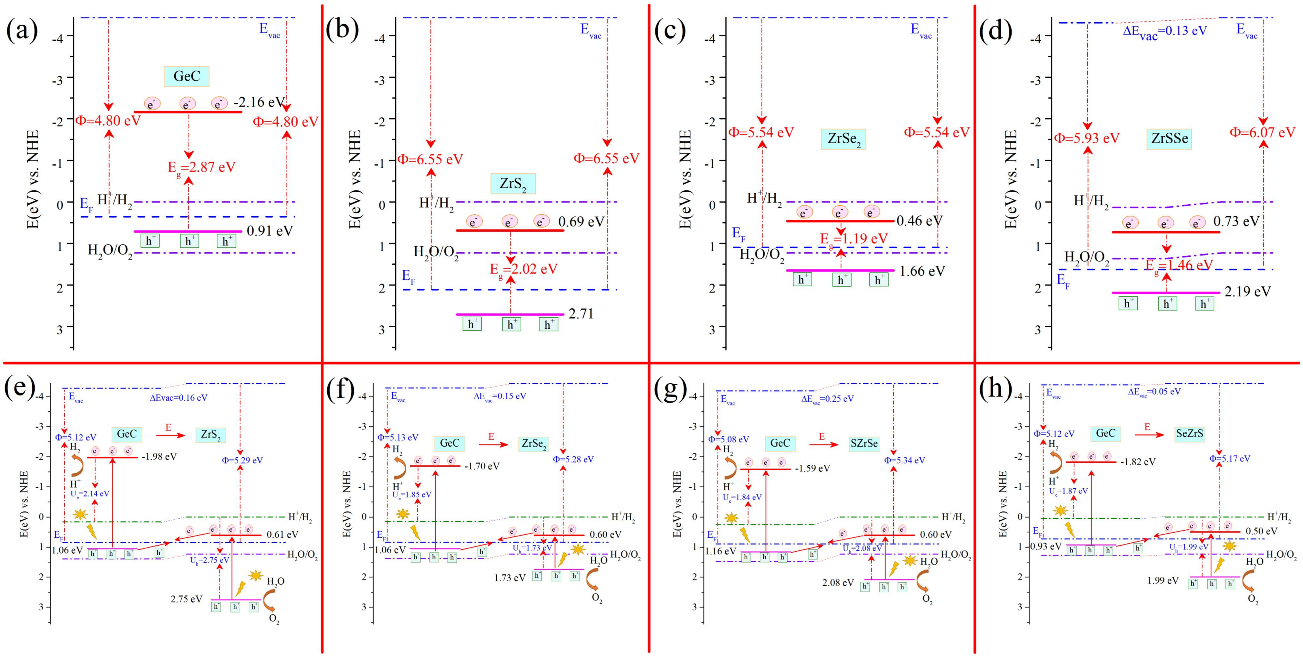

Fig. S5 Schematic diagrams of the photocatalytic mechanisms for (a) GeC, (b) ZrS_2, (c) $ZrSe_2$, (d) ZrSSe, (e) GeC/ZrS_2, (f) GeC/$ZrSe_2$, (g) GeC/SZrSe and (h) GeC/SeZrS versus NHE.

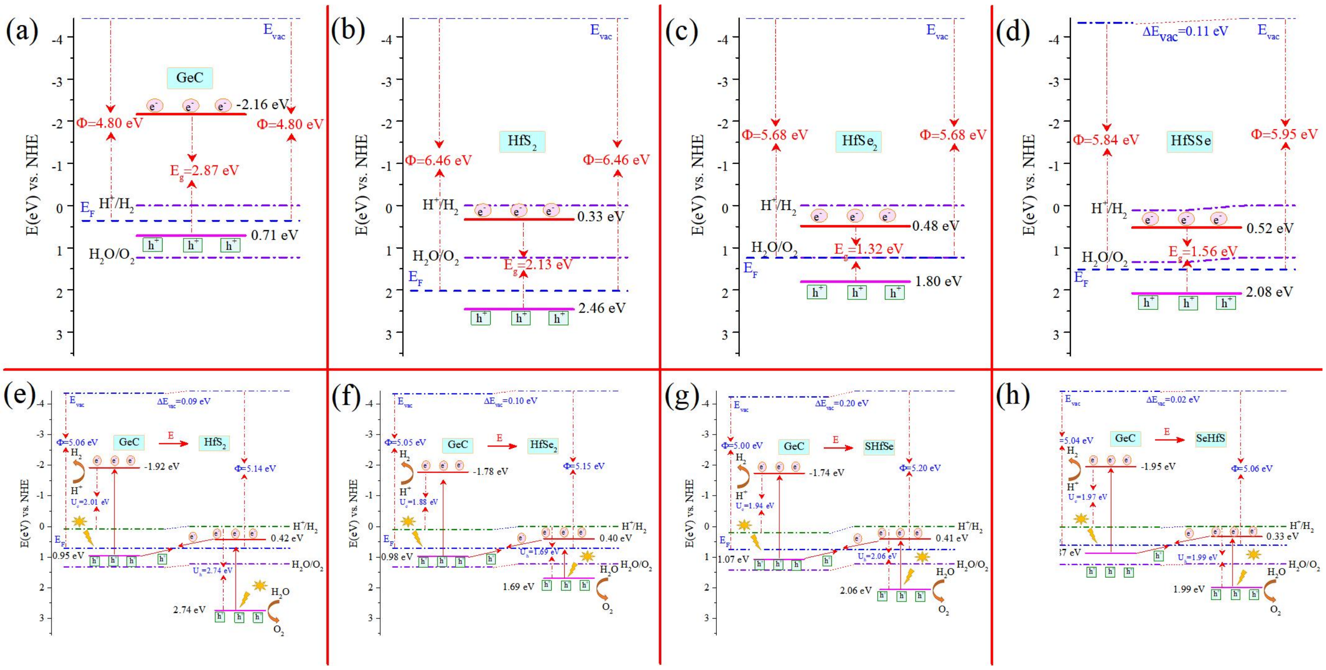

Fig. S6 Schematic diagrams of the photocatalytic mechanisms for (a) GeC, (b) HfS_2, (c) $HfSe_2$, (d) HfSSe, (e) GeC/HfS_2, (f) GeC/$HfSe_2$, (g) GeC/SHfSe and (h) GeC/SeHfS versus NHE.

Table S1. POSCAR file for the optimized GeC/ZrS_2.

```
GeC/ZrS2
   1.00000000000000
     6.4263000487999999    0.0000000000000000    0.0000000000000000
    -3.2131500244000000    5.5653390945999996    0.0000000000000000
     0.0000000000000000    0.0000000000000000   24.3579998015999983
   Ge    C     Zr    S
   4     4     3     6
Direct
  0.3336907235635558  0.1665485584538447  0.3574160184367035
  0.8334600054281847  0.1671547568850910  0.3574166290836183
  0.3333331574623474  0.6666746924673291  0.3570352560724598
  0.8328530539169345  0.6663180052385993  0.3574176543021175
  0.1671669901114386  0.3333623552984661  0.3561556491197173
  0.6666675069844237  0.3333405004434898  0.3566231668251763
  0.1661837571265795  0.8328473773287173  0.3561580560857663
  0.6666463962222478  0.8338188800067435  0.3561579801715872
 -0.0000012088375214 -0.0000068546523914  0.5550816711282811
  0.6666664373078877  0.3333260681023428  0.5536321042572330
  0.3333309137513146  0.6666618093617241  0.5553427230603460
  0.0012836210014878  0.3332139086018826  0.4956557362596699
  0.6667800795857844  0.6680546731254671  0.4956556400028980
  0.3319375052310346 -0.0012911395437037  0.4956561483065306
 -0.0012280186776452  0.6652975177646752  0.6135914804554082
  0.6665346549327436  0.0012198785233754  0.6135919133838682
  0.3346945118891985  0.3334590995943437  0.6135922280485887
```

Table S2. POSCAR file for the optimized GeC/$ZrSe_2$.

```
GeC/ZrSe2
   1.00000000000000
     6.5265002251000004    0.0000000000000000    0.0000000000000000
    -3.2632501125000002    5.6521149926999996    0.0000000000000000
     0.0000000000000000    0.0000000000000000   24.8211994171000008
   Ge   C    Zr   Se
     4     4     3     6
Direct
  0.3336611287477901  0.1665091079068270  0.3363510501094507
  0.8334759143039511  0.1671219706587174  0.3363470142919069
  0.3333451076366500  0.6666500192248377  0.3359593776660922
  0.8328721620361005  0.6663306593400236  0.3363473448407826
  0.1673404688289752  0.3334196251436874  0.3353035093248311
  0.6666826639117349  0.3333401540921644  0.3352879075974561
  0.1661049265895872  0.8326682487594197  0.3352995100076893
  0.6665853252596812  0.8339288657989488  0.3352977676422521
 -0.0000054234206759  0.0000041291801836  0.5389458219904640
  0.6666613804221367  0.3333369249774529  0.5398092719407274
  0.3333293057335328  0.6666696411334593  0.5395958314886377
 -0.0004515961769814  0.3336855542830213  0.4751659745121444
  0.6663056409997974  0.6658790681813264  0.4751657938425334
  0.3341138654358233  0.0004464432224613  0.4751655111962881
  0.0003268301572408  0.6672809511464477  0.6037995319285266
  0.6669368002609023 -0.0003314047944669  0.6037993376203002
  0.3327155862737509  0.3330601287454864  0.6037996589999336
```

Table S3. POSCAR file for the optimized GeC/SZrSe.

```
GeC/SZrSe
   1.00000000000000
     6.4770002365000003    0.0000000000000000    0.0000000000000000
    -3.2385001183000002    5.6092467451000001    0.0000000000000000
     0.0000000000000000    0.0000000000000000   24.5333003998000017
   C    Ge   Zr   Se   S
     4     4     3     3     3
Direct
  0.3333166798035085  0.1661782280037082  0.3372582393256638
  0.8337950316431144  0.1672283884668150  0.3372551297757544
  0.3333334546890229  0.6666854692791870  0.3376325832024125
  0.8328062531327948  0.6666819520492953  0.3372478080199395
  0.1671115050082983  0.3336884159158595  0.3384022464294916
  0.6666314060602363  0.3333607024224992  0.3380211238436031
  0.1665112102223299  0.8328813331941751  0.3384000270300098
  0.6663406424350371  0.8335121139727585  0.3384027525224177
  0.0000183911076775 -0.0000275977435259  0.5329900762922861
  0.3333553358657730  0.6666405679961971  0.5319025545521225
  0.6666862363485310  0.3333128018394340  0.5333455516291921
  0.3332615215858989 -0.0012383998977277  0.5995078399358983
  0.6655473475477944  0.6667295684989365  0.5995064548006397
  0.0012325426581006  0.3344312404287620  0.5995088836672997
  0.6677258660289918  0.0010503260996143  0.4760164552025297
 -0.0010435390385809  0.6666229759894071  0.4760152522983868
  0.3333702019014677  0.3322620004846012  0.4760169344723446
```

Table S4. POSCAR file for the optimized GeC/SeZrS.

```
GeC/SeZrS
   1.00000000000000
     6.4770002365000003    0.0000000000000000    0.0000000000000000
    -3.2385001183000002    5.6092467451000001    0.0000000000000000
     0.0000000000000000    0.0000000000000000   24.6333007813000009
   Ge   C    Zr   Se   S
     4     4     3     3     3
Direct
  0.3335842722525126  0.1664913854832828  0.3529976865312253
  0.8334826144605515  0.1670955959677137  0.3529936625007304
  0.3333327456157679  0.6666621209883505  0.3525595048938448
  0.8328660042545478  0.6663712825027337  0.3529933761747995
  0.1672547894975669  0.3333602687028016  0.3519827476043988
  0.6666385459562794  0.3333119978202025  0.3522039987912156
  0.1660703063072050  0.8327018125681526  0.3519783631222519
  0.6666263818998582  0.8338935284333722  0.3519755421119669
  0.6666874066505840  0.3333451482576849  0.5595190073233279
  0.3333518288079161  0.6666791788362285  0.5588480072741813
  0.0000117891456812  0.0000086770012061  0.5585551493228013
  0.6666275768812872  0.6657041697187023  0.4924739468816924
  0.3343237693487513  0.0009302222610222  0.4924747243282532
 -0.0009020768777488  0.3334028013412255  0.4924747052526892
  0.6665922171633250 -0.0006909868257184  0.6153805115787139
  0.3327270046329441  0.3334355224102153  0.6153789697640659
  0.0007249110029666  0.6672973615328204  0.6153801155438299
```

Table S5. POSCAR file for the optimized GeC/HfS_2.

```
GeC/HfS2
   1.00000000000000
     6.3916001319999998    0.0000000000000000    0.0000000000000000
    -3.1958000659999999    5.5352880851000004    0.0000000000000000
     0.0000000000000000    0.0000000000000000   24.3160991668999991
   Ge   C    Hf   S
     4     4     3     6
Direct
  0.3335337295701139  0.1672928958560853  0.3382364120754396
  0.8327041346043706  0.1662170101506327  0.3382367985630532
  0.3333303770371774  0.6666668185549459  0.3379542919003004
  0.8337784819413464  0.6664661559593826  0.3382363087285211
  0.1659902508781516  0.3333519022229954  0.3373185070176114
  0.6666642431068576  0.3333359410873920  0.3376169516888713
  0.1673812572717810  0.8340273962595690  0.3373163630992321
  0.6666452014359103  0.8326067051673425  0.3373052249580093
 -0.0000034057980062  0.0000037387772523  0.5394064852989122
  0.3333325673589209  0.6666681965429408  0.5395490600360462
  0.6666610501813648  0.3333395996851257  0.5391298237316313
  0.0001907923272678  0.6668954614054353  0.4808565330487365
  0.3331081931746305  0.3333087134423745  0.4808575230575761
  0.6666919263332689 -0.0001914477853233  0.4808562040213927
 -0.0001991475456061  0.3333789005660444  0.5978651392949147
  0.3335693962518876  0.0002016154806407  0.5978646287128382
  0.6666210388705596  0.6664304836271603  0.5978635087669123
```

Table S6. POSCAR file for the optimized GeC/$HfSe_2$.

```
GeC/HfSe2
   1.00000000000000
     6.4988999367000000    0.0000000000000000    0.0000000000000000
    -3.2494499683000000    5.6282124417999997    0.0000000000000000
     0.0000000000000000    0.0000000000000000   24.7724990845000015
   Ge   C    Hf   Se
     4     4     3     6
Direct
  0.3335215792087528  0.1668777758968527  0.3257018965324913
  0.8330613188562863  0.1666966143715246  0.3257052574169196
  0.3333395344955231  0.6666513880215199  0.3254616804331907
  0.8333402184535575  0.6664602802109838  0.3256980233485445
  0.1662872052979044  0.3333863475105012  0.3248953758870685
  0.6666591863758152  0.3333379615194725  0.3251710772462378
  0.1670815320903704  0.8336776632803787  0.3248895668798091
  0.6666081763426904  0.8329422314553067  0.3248915255016342
  0.0000099731002642 -0.0000033723553294  0.5296126651597810
  0.3333444475251814  0.6666655160342448  0.5301053203349912
  0.6666801947312276  0.3333283828170234  0.5303916149210685
 -0.0005247816755569  0.6658430900286578  0.4663373255108810
  0.3341627713041622  0.3336168750685011  0.4663400621485318
  0.6663942346842017  0.0005226257486420  0.4663371309795925
  0.0004674105056577  0.3331268612519805  0.5937746977700976
  0.3326793886961948 -0.0004567420652488  0.5937734531925072
  0.6668876970077630  0.6673265882049837  0.5937734047366771
```

Table S7. POSCAR file for the optimized GeC/SHfSe.

```
GeC/SHfSe
   1.00000000000000
     6.4442000389000000     0.0000000000000000     0.0000000000000000
    -3.2221000195000000     5.5808409407999999     0.0000000000000000
     0.0000000000000000     0.0000000000000000    24.4892997741999991
   Ge   C    Hf   Se   S
     4     4     3     3     3
Direct
  0.3335794789003109  0.1664907333665439  0.3283554228841737
  0.8335071229251771  0.1670922112354869  0.3283480571692937
  0.3333471277728719  0.6666532141030562  0.3280853593685802
  0.8329370339091339  0.6664238053937356  0.3283526617130385
  0.1671353538753974  0.3333508670749967  0.3276277622514882
  0.6666673945113032  0.3333456747003249  0.3277237349196123
  0.1662450579294754  0.8328604313845043  0.3276235659773017
  0.6666555016240477  0.8337700949428504  0.3276286014635497
 -0.0000063683752092  0.0000004723175394  0.5244340463397963
  0.6666642342764446  0.3333340104436662  0.5238918730531166
  0.3333236117688096  0.6666674703101766  0.5247450741692927
  0.6667444409001323  0.0006222625219085  0.5903592665488221
  0.3338501986799751  0.3332440889340435  0.5903597337175862
 -0.0006290931087639  0.6661415208429390  0.5903588449386388
  0.3329063258047750 -0.0005139325015828  0.4680689588403638
  0.0005062225387940  0.3334263552952534  0.4680688892820395
  0.6665664430673218  0.6670908066345533  0.4680683233632955
```

Table S8. POSCAR file for the optimized GeC/SeHfS.

```
GeC/SeHfS
   1.00000000000000
     6.4442000389000000     0.0000000000000000     0.0000000000000000
    -3.2221000195000000     5.5808409407999999     0.0000000000000000
     0.0000000000000000     0.0000000000000000    24.5893001556000002
   Ge    C     Hf    Se    S
     4     4     3     3     3
Direct
  0.3335351919445925  0.1669335064696862  0.3349837741388297
  0.8330626084313267  0.1665762350191344  0.3349887107313683
  0.3333338741230267  0.6666540405533194  0.3347602629237764
  0.8334229033003153  0.6664723394029319  0.3349885393268225
  0.1662555560011599  0.3333703195706868  0.3341596214106456
  0.6666715759199622  0.3333311697724672  0.3343761981981241
  0.1671244110016911  0.8337439813316821  0.3341614190307105
  0.6666266632920849  0.8328754921490118  0.3341615533902403
 -0.0000056669860492  0.0000041697158474  0.5434304229003744
  0.3333298586074777  0.6666707591441832  0.5438134753094782
  0.6666643139816548  0.3333389057889066  0.5443782597434387
 -0.0008084402584146  0.6655858214406913  0.4781122381161612
  0.3344157311061188  0.3336186346079985  0.4781124730366377
  0.6663826457630151  0.0008082804264685  0.4781121789662199
  0.0006099850842647  0.3332481247950072  0.5999583237289982
  0.3326239976332103 -0.0006058643017625  0.5999559442875718
  0.6667548780545607  0.6673741711137366  0.5999566207605660
```
